# Association Between Emotional Intelligence and Stress Management in Hemodialysis Patients

**DOI:** 10.3390/clinpract15080153

**Published:** 2025-08-19

**Authors:** Orchan Impis, Afroditi Zartaloudi, Eirini Grapsa, Georgia Gerogianni

**Affiliations:** 1 Department of Nursing, University of West Attica, 12243 Athens, Greece; oimpis@uniwa.gr (O.I.); azarta@uniwa.gr (A.Z.); 2 Department of Nephrology, Aretaieio Hospital, National and Kapodistrian University of Athens, 11528 Athens, Greece; egrapsa@aretaieio.uoa.gr

**Keywords:** emotional intelligence, coping strategies, management of stress, hemodialysis

## Abstract

**Background:** Emotional intelligence refers to individuals’ ability to recognize and manage their own emotions as well as those of others, playing a crucial role in stress management. This study aimed to investigate the relationship between different dimensions of emotional intelligence and stress management strategies in patients undergoing hemodialysis. **Methods:** In this cross-sectional study, 468 patients on hemodialysis completed the (i) Wong and Law Emotional Scale (WLEIS) and (ii) Trait Emotional Intelligence Questionnaire Short Form (TEIQue-SF) for the assessment of emotional intelligence as an emotional ability or as a personality trait, respectively; (iii) the Brief COPEQuestionnaire (Brief- COPE) for the assessment of stress management strategies; and (iv) a questionnaire about demographic characteristics. Spearman’s correlations coefficients were used to explore associations between two continuous variables. Multiple linear regression analysis was used with Brief-COPE dimensions as the dependent variable. **Results:** High levels of emotionality were associated with an active approach to coping with stress (*p* = 0.018), while increased well-being and high regulation of emotions were associated with decreased behavioral disengagement (*p* < 0.001). Moreover, high emotional appraisal of others was linked to an increased use of humor (*p* = 0.042), while self-control and use and regulation of emotions were associated with decreased expression of negative feelings (*p* < 0.001). **Conclusions:** The current findings suggest potential links between emotional intelligence and stress management strategies in patients undergoing hemodialysis.

## 1. Introduction

Emotional intelligence is a combination of emotional and social skills that enables individuals to recognize, understand, and manage emotions; build relationships; adapt to change; solve personal and social problems; and effectively cope with everyday challenges [1,2]. It involves a range of skills that enable individuals to identify, assess, and regulate both their own emotions and those of others [3]. Emotional intelligence promotes adaptive coping, leading to an improved health-related quality of life [4], since it enhances individuals’ ability to manage and utilize emotions, which in turn impacts both their physical and mental well-being [5].

Emotional intelligence has been conceptualized from various theoretical perspectives, most commonly as either a stable personality trait or a set of emotional abilities. The Trait Emotional Intelligence model of Petrides and Furnham focuses on how individuals perceive and interpret their own emotional experiences [6] and refers to a set of self-perceived emotional traits that reflect how a person evaluates their own emotional and social abilities [7]. In contrast, the ability model of Mayer and Salovey emphasizes individuals’ abilities to recognize and manage emotions in themselves and others [8]. According to this model, emotional intelligence includes four main skills: accurately recognizing emotions, using emotions to support thinking, understanding emotions, and controlling emotions. This ability-based approach considers emotional intelligence as one part of overall human intelligence [8].

Given the conceptual differences between the above two models of emotional intelligence, the present study employed both the Wong and Law Emotional Intelligence Scale (WLEIS) [9], based on the model of Mayer and Salovey, and the Trait Emotional Intelligence Questionnaire Short Form (TEIQue-SF), based on the trait model of Petrides and Furnham, in order to assess both patients’ perceived emotional experiences and their ability to recognize and manage emotions in themselves and others.

Research has shown that emotional intelligence plays a crucial role in coping with stress by reducing negative emotions such as anxiety, anger, and low self-esteem while fostering positive emotions like empathy, friendliness, and self-confidence, thereby enhancing individuals’ self-efficacy [10]. A similar study confirms that high levels of emotional intelligence are linked to reduced anxiety in patients undergoing hemodialysis [11].

Patients undergoing hemodialysis often experience heightened stress due to challenges in adhering to treatment restrictions. The most common stressors include dietary and fluid limitations, changes in family roles, frequent hospital admissions, reduced participation in daily activities, uncertainty about the future, increased dependence on healthcare professionals, job loss, and impaired sexual function [12,13]. They also face challenges with taking vacations, spending long hours on dialysis, fatigue, financial difficulties, reduced social life, body image concerns, and decreased functional ability [14].

Although several studies have examined emotional intelligence or management of stress in the Greek population, few have investigated the relationship between emotional intelligence and stress management strategies specifically in patients undergoing hemodialysis in Greece. Using both measures in the present study allows for a more comprehensive assessment of emotional intelligence, capturing complementary but distinct dimensions that may relate differently to coping strategies in hemodialysis patients.

The aim of this study was to investigate the association between dimensions of trait and ability emotional intelligence and stress management strategies in patients undergoing hemodialysis.

## 2. Materials and Methods

### 2.1. Study Sample

Inclusion criteria were as follows: age between 18 and 85 years, undergoing hemodialysis for at least three months, and the ability to speak, read, and write in Greek. Exclusion criteria included insufficient language ability, age over 85 years, cognitive impairment, and drug or alcohol abuse. Cognitive impairment was assessed based on medical records and clinical evaluations conducted by the treating nephrologist and nursing team. Patients with documented cognitive decline or those unable to reliably complete the questionnaires were excluded.

A total of 887 individuals were approached to participate in the study, of whom 80 refused to participate despite meeting the inclusion criteria. Consequently, questionnaires were distributed to 807 people. Of these, 620 individuals returned the questionnaires (response rate 76.85%), of which 468 (57.99%) were valid, while 152 (18.83%) were rejected due to incomplete answers, and 187 (23.17%) questionnaires were not returned.

This study was conducted from November 2023 to July 2024. Data collection was conducted through interviews using the following questionnaires: (i) the Wong and Law Emotional Intelligence Scale (WLEIS) to assess emotional intelligence as an ability, (ii) the Trait Emotional Intelligence Questionnaire Short Form (TEIQue-SF) to evaluate emotional intelligence as a personality trait, (iii) the Brief COPE Questionnaire to assess coping strategies employed by individuals to manage stress and problems, and (iv) a questionnaire on demographic characteristics.

### 2.2. Instruments

#### 2.2.1. Wong and Law Emotional Intelligence Scale (WLEIS)

The Wong and Law Emotional Intelligence Scale (WLEIS), developed by Wong and Law, consists of 16 items rated on a seven-point Likert scale (1 = strongly disagree to 7 = strongly agree). It is a self-report measure that assesses four dimensions of emotional intelligence [9]. The dimensions of the scale are (i) understanding the emotions of others, (ii) self-evaluation of emotions, (iii) use of emotions, and (iv) emotion regulation. Higher scores indicate higher levels of emotional intelligence. The scale demonstrates good validity and internal consistency in the Greek population, with a Cronbach’s alpha of 0.90. The Greek version of the Wong and Law Emotional Intelligence Scale (WLEIS) is a reliable and valuable tool for research involving the Greek population [15].

#### 2.2.2. Trait Emotional Intelligence Questionnaire Short Form (TEIQue-SF)

The short form of the Trait Emotional Intelligence Questionnaire (TEIQue-SF) consists of 30 items and was developed as a reliable instrument to assess emotional intelligence as a trait. The questionnaire includes two items for each of the fifteen subscales from the full TEIQue, with responses rated on a seven-point Likert scale (1 = strongly disagree to 7 = strongly agree) [16]. Higher scores indicate greater levels of emotional intelligence. The 15 subscales assess various aspects, including understanding of emotions, expression of emotions, social awareness, motivation and self-esteem, relationships, emotion management, empathy, happiness and optimism, assertiveness, adaptability and reduced impulsivity, emotion regulation, and stress control. The full version of the TEIQue questionnaire has been translated into Greek [17] and is available from the London Psychometric Laboratory free of charge for academic research. It demonstrates good validity and internal consistency in the Greek population [18].

#### 2.2.3. Brief COPE Questionnaire (Brief-COPE)

The Brief COPE Questionnaire (Brief-COPE) is a psychometric instrument that assesses the strategies individuals use to cope with stress and problems [19]. The scale consists of 28 items assessing fourteen coping strategies, with responses rated on a four-point Likert scale ranging from 1 (“I don’t do it at all”) to 4 (“I do it a lot”). Each of the 14 subscales includes two items and measures the following coping strategies: active coping, acceptance, planning, seeking emotional support, seeking practical support, humor, religion, denial, substance use, positive reappraisal, behavioral disengagement, self-avoidance (avoidance), self-blame, and venting (expressing negative emotions). The scale has been translated into Greek. Higher scores in each dimension indicate a stronger tendency to use the corresponding coping strategy. The Greek version of the scale demonstrates adequate psychometric properties, satisfactory construct validity, and reliable internal consistency [20]. It has a Cronbach’s a value of 0.86, indicating good reliability in the Greek population [21].

### 2.3. Statistical Analysis

Quantitative variables were expressed as mean values (SD) or as median (IQR), while qualitative variables were expressed as absolute and relative frequencies. Spearman’s correlation coefficients were used to explore the association of two continuous variables [22], and statistical significance was determined using the Bonferroni correction. Correlations with *p*-values less than 0.001 were considered significant. Multiple hierarchical linear regression analysis was used with the Brief-COPE dimensions as the dependent variable. In the first step, participants’ demographic and clinical characteristics were entered. In the second step, dimensions of emotional intelligence were entered in a stepwise method (*p* for entry 0.05, *p* for removal 0.10), since they were highly correlated with each other. Scales TEIQue-SF and WEILS were entered in the analysis alternately due to being highly correlated with each other. Adjusted regression coefficients (β) with standard errors (SE) were computed from the results of the linear regression analyses. Log transformations were used for the dependent variable due to lack of normal distribution. VIF was used to assess multicollinearity among the independent variables. Residual plots were used to validate assumptions about normality and homoscedasticity in the regression models. The autocorrelation of the residuals was tested using the Durbin–Watson statistic. All reported *p*-values are two-tailed. Statistical significance was set at *p* < 0.05, and analyses were conducted using SPSS statistical software (version 26.0) [22,23].

### 2.4. Ethics

Data collection was conducted after receiving approval from the Ethics Committee of the University of West Attica (Number of approval: 28th/03-11-2023). The study participants were informed about the purpose of the study, the procedures involved, and the confidentiality of their data. The study was conducted in accordance with the Declaration of Helsinki {1989}.

## 3. Results

### 3.1. Participant Characteristics

The sample consisted of 468 patients on hemodialysis with a mean age of 63.9 years (SD = 13.1 years). Of the whole sample, 61.3% were men, and nearly all (96.4%) were of Greek nationality. A total of 75.4% resided in the Attica region, 43.6% were middle school/high school graduates, and 28.2% were graduates of a university or technological institute. Additionally, 63.5% were married, and 81.8% had children, with a median of two children. Only 20.1% lived alone, 69.7% were retirees, and 9% rated their financial status as very good to excellent.

The median duration of hemodialysis was 36 months (IQR: 16–72 months), and 95.5% underwent hemodialysis three times a week. The mean duration of each hemodialysis session was 3.8 h (SD = 0.4). Concerning vascular access, 65.4% of the participants had a fistula, 24.2% had a catheter, and 9.4% had a graft. The majority of patients (86.1%) had public insurance coverage, 38.5% were candidates for a kidney transplant, and 7.9% had received a transplanted. Also, 73.7% suffered from comorbidities (Table 1).

### 3.2. Association of Wong and Law Emotional Scale (WLEIS) and Trait Emotional Intelligence Questionnaire Short Form (TEIQue-SF) with Brief COPEQuestionnaire (Brief-COPE)

From the correlation analysis between stress management (Brief-COPE) and the emotional intelligence scales WLEIS and, TEIQue-SF it was found that better self- and other-emotion appraisal, better use and regulation of emotions, and higher emotionality, self-control, well-being, and sociability were associated with more active positive coping and reduced behavioral disengagement (only sociability was not significantly correlated with behavioral disengagement).

Better self- and other-emotion appraisal and overall emotional intelligence (TEIQue-SF) were associated with reduced substance use as a coping strategy for stress. Better use and regulation of emotions and greater self-control were linked to reduced seeking of support.

Additionally, use and regulation of emotions were significantly and negatively associated with the expression of negative emotions, as were emotionality, self-control, well-being, and the overall TEIQue-SF scale (Table 2).

### 3.3. Multiple Linear Regression Analyses with Dimensions of Brief-COPE Scale as Dependent Variables

Active positive coping was associated with educational level, the dimensions of emotionality and well-being, and the use of emotions. Graduates of postgraduate/PhD programs demonstrated a more active and positive approach to coping with stress and problems compared to elementary school graduates. Higher levels of emotionality and greater well-being were associated with a more active approach to coping with stress and problems.

Additionally, increased well-being, as well as higher self-control and better regulation of emotions, were associated with decreased behavioral disengagement. Regarding substance abuse, it was negatively influenced by well-being and emotional self-appraisal.

Seeking support was found to be associated with educational level, self-control, and the regulation of emotions. Graduates of middle school and high school, as well as postgraduate/doctoral graduates, were less likely to seek support compared to elementary school graduates. Also, greater self-control and better regulation of emotions were associated with reduced help-seeking behavior. Sex and age were the two factors that affected religion.

Women and older people turned more to religion. Results were similar for the dimension of avoidance; women were more likely to avoid dealing with problems compared to men.

The humor dimension was associated with age, well-being, and emotional appraisal of others. Older age was associated with a reduced use of humor in coping with stress and problems, whereas greater well-being and better emotional appraisal of others were linked to an increased use of humor. Finally, comorbidities were associated with a greater expression of negative feelings, while self-control, well-being, and use and regulation of emotions were associated with decreased expression of negative feelings (Table 3).

Regression diagnostics were conducted for all models. Visual inspections using histograms and Q-Q plots indicated no violations of the assumptions related to the residuals. VIF ranged from 1.12 to 2.40, remaining below the commonly accepted threshold of 5, suggesting that multicollinearity was not a concern. Additionally, the Durbin–Watson statistic did not exceed the value of 2 in any model, indicating no evidence of autocorrelation in the residuals.

## 4. Discussion

This study showed that higher levels of emotionality and greater well-being were associated with a more active approach to coping with stress and problems, while increased well-being, as well as higher self-control and better regulation of emotions, were associated with decreased behavioral disengagement. This may be attributed to the fact that individuals with high emotional awareness are able to effectively recognize and manage their emotions during stressful situations, allowing them to adjust their thoughts and behaviors in socially appropriate ways [24]. It is important to consider that individuals with strong emotional regulation are more likely to manage distress with a positive attitude and effective coping strategies. This, in turn, enhances their resilience, fosters empathy, and enables them to handle life’s pressures without becoming overwhelmed. Consequently, people with high emotional intelligence often perceive themselves as more resilient and better equipped to deal with challenges and negative experiences [24]. These findings align with those of a similar study involving 138 patients undergoing chronic hemodialysis, which found that higher emotional intelligence was significantly associated with better perceived general and emotional health, particularly among women [5].

Moreover, the results of this study showed that high overall emotional intelligence among dialysis patients was associated with reduced substance use as a coping strategy for stress. A possible explanation for this finding is that emotional intelligence plays a protective role against negative life experiences and is associated with the frequent use of adaptive coping strategies, thereby reducing the psychological impact of issues such as anxiety and depression [25]. Similarly, individuals with high levels of emotional intelligence are less likely to engage in unhealthy coping behaviors such as substance use and are better able to resist social pressure to consume alcohol and drugs. Specifically, higher emotional intelligence enhances self-control and emotion regulation, which in turn support better decision-making and increase the likelihood of using adaptive coping strategies [25]. It is important to note that poor emotion regulation can lead to substance use by impairing an individual’s ability to control their behavior in emotionally intense situations [26].

Additionally, the findings of this study indicated that greater well-being and better emotional appraisal of others were linked to an increased use of humor. It is important to consider that emotional intelligence is associated with a greater use of positive emotion regulation strategies [27]. Humor is an effective positive coping strategy for alleviating stress [28], promoting a positive mood, encouraging optimistic thoughts, and fostering a supportive social environment [29,30]. A similar study involving 63 patients on hemodialysis found that humor therapy was effective in reducing anxiety [31].

The present study also showed that self-control, well-being, and use and regulation of emotions were associated with decreased expression of negative feelings. This may be explained by the role of trait emotional intelligence in reducing stress and negative emotional responses [32]. Therefore, emotional intelligence plays a crucial role in emotional regulation, enhancing an individual’s ability to manage stressful situations by promoting coping strategies that reduce negative emotions and sustain positive ones [33]. This finding aligns with a similar study involving 289 individuals with lung cancer, which found that those with higher emotional intelligence had a greater ability to cope with negative emotions related to cancer symptoms and treatment [32].

Also, greater self-control and better regulation of emotions were associated with reduced help-seeking behavior. Individuals with high emotional intelligence may rely more on internal coping strategies and self-regulation, rather than seeking external support [34]. This tendency is influenced by cultural norms, especially in societies where emotional expression or seeking help is discouraged or stigmatized. A related study found that cultural values such as collectivism, power distance, and uncertainty avoidance significantly shape how individuals manage emotional situations. In these contexts, emotionally intelligent individuals may avoid burdening others or showing vulnerability, preferring coping strategies that maintain social harmony and align with culturally preferred conflict-handling styles such as avoidance, collaboration, or accommodation [35].

This study also showed that graduates of postgraduate/PhD studies demonstrated a positive approach to coping with stress and problems. One possible explanation is that higher education may facilitate the development of cognitive and informational resources that support effective self-management. This level of knowledge is considered a key element in enhancing self-management, as it enables a clear understanding of medical conditions [13]. Previous research involving 256 patients on hemodialysis has suggested that higher educational levels are often linked to greater psychological resilience, possibly due to better illness comprehension, more adaptive coping mechanisms, and increased access to informational and healthcare resources [36]. However, a study involving 129 dialysis patients showed that those with a moderate level of education could also experience high levels of emotional well-being, indicating that education alone does not define coping capacity [37]. These findings suggest that while education may be a facilitating factor, individualized support remains essential across all educational groups.

Furthermore, the current study revealed that older age was associated with a reduced use of humor in coping with stress and problems. This can be viewed in the context of comorbid diseases, which are common among the elderly and often lead to pain, fatigue, social isolation, anxiety, depression, fear, uncertainty, hopelessness, frustration, and anger [38]. In a similar study involving 403 elderly individuals, elevated anxiety levels were observed, and more than half of the participants demonstrated inadequate stress management abilities [39].

Moreover, the findings of the present study indicated that women were more likely to avoid dealing with problems compared to men. It is important to consider that individuals who rely on this coping strategy often deny their illness or underestimate its severity, which can lead to poor treatment adherence [40]. Similarly, another study found that women scored high in self-distraction, denial, and venting, all of which were strongly associated with increased levels of distress. It is possible that gender-related stereotypes about emotional expression influence how men and women communicate their feelings [41].

Thus, personalized interventions, such as cognitive-behavioral therapy for women and emotion-focused strategies, including the use of humor for older adults, can enhance emotional flexibility and improve psychological well-being. Clinicians can incorporate gender- and age-sensitive strategies into psychological support programs, improving emotional adjustment, treatment engagement, and overall quality of care for patients on hemodialysis.

The cross-sectional design does not allow causal inferences, and the use of self-reported measures may introduce response biases. However, it should be noted that the cross-sectional design is appropriate for an initial exploration of relationships between variables but has inherent limitations regarding causality. The potential for non-response bias (from ~23% who did not return the questionnaire) should be emphasized. Patients experiencing higher levels of stress may have been less willing or able to participate in the study, potentially introducing selection bias. This could mean that the sample underrepresents individuals with the greatest stress-related challenges, which may limit the generalizability of the findings to the broader dialysis population.

The current findings suggest potential links between emotional intelligence and stress management strategies. Future studies employing longitudinal or mixed-methods designs are needed to explore these associations over time and to clarify possible causal pathways. Additionally, qualitative approaches may offer deeper insight into the subjective experiences and contextual factors shaping coping behaviors in patients undergoing hemodialysis.

## 5. Conclusions

This cross-sectional study identified significant associations between emotional intelligence and various coping strategies among patients undergoing hemodialysis, including increased use of active and positive coping, reduced behavioral disengagement, and greater reliance on humor. These findings suggest that emotional intelligence may influence how patients manage the emotional challenges associated with their condition. However, due to the cross-sectional design, causal relationships cannot be established. Further longitudinal and interventional studies are needed to determine whether enhancing emotional intelligence can support psychological resilience and improve emotional well-being in this population.

## Figures and Tables

**Table 1:** Sample demographic and clinical characteristics.

			N	%
Gender	Male		287	61.3
	Female		179	38.2
	Other		2	0.4
Age			63.9 (13.1)	
Ethnicity	Greek		451	96.4
	Other		17	3.6
Residence in Attica region	No		115	24.6
	Yes		353	75.4
Educational level	Primary school		91	19.4
	Middle school–High school		204	43.6
	Technological Educational Institute/University		132	28.2
	Master’s degree–PhD		41	8.8
Married/living together	No		161	34.4
	Yes		307	65.6
Children	No		85	18.2
	Yes		383	81.8
Living alone	No		374	79.9
	Yes		94	20.1
Employment status	Public servant		14	3.0
	Self-employed		30	6.4
	Unemployed		21	4.5
	Private-sector employee		43	9.2
	Household		32	6.8
	Student		2	0.4
	Pensioner		326	69.7
Financial status	Poor		42	9.0
	Moderate		217	46.4
	Good		167	35.7
	Very good		35	7.5
	Excellent		7	1.5
Months of hemodialysis, Median (IQR)			36.0 (16–72)	
Session duration, Mean (SD)			3.8 (0.4)	
Vascular access		Fistula	306	65.4
		Graft	44	9.4
		Catheter	114	24.2
Insurance		Public	403	86.1
		Private	17	3.6
		Both	35	7.5
		Uninsured	13	2.8
Are you a candidate for a kidney transplant?		No	288	61.5
		Yes	180	38.5
Have you had a kidney transplant in the past?		No	431	92.1
		Yes	37	7.9
Comorbidities		No	124	26.3
		Yes	345	73.7

**Table 2:** Spearman’s correlation coefficients between dimensions of Brief-COPE, WEILS, and TEIQue-SF.

Brief-COPE		Active Positive Coping	Behavioral Disengagement	Substance Abuse	Seeking Support	Religion	Humor	Avoidance	Express Negative Feelings
**WLEIS**	Emotion self-appraisal	0.27 *	−0.29 *	−0.19 *	−0.14	−0.02	0.07	0.02	−0.12
	Emotion appraisal of others	0.27 *	−0.20 *	−0.16 *	−0.08	0.03	0.12	0.10	−0.04
	Use of emotion	0.34 *	−0.28 *	−0.13	−0.16 *	0.01	0.08	0.05	−0.19 *
	Regulation of emotion	0.22 *	−0.31 *	−0.13	−0.18 *	−0.01	0.03	−0.02	−0.24 *
**TEIQue-SF**	Emotionality	0.24 *	−0.29 *	−0.14	−0.06	0.04	0.12	−0.03	−0.22 *
	Self-control	0.24 *	−0.34 *	−0.06	−0.21 *	−0.09	0.05	−0.09	−0.29 *
	Well-being	0.32 *	−0.41 *	−0.11	−0.07	0.01	0.12	−0.09	−0.26 *
	Sociability	0.20 *	−0.11	−0.01	−0.03	−0.06	0.08	0.06	−0.04
	Total TEIQue-SFscore	0.35 *	−0.41 *	−0.10 *	−0.13	−0.02	0.14	−0.05	−0.30 *

* *p* < 0.001.

**Table 3:** Multiple linear regression analyses with dependent variables the dimensions of Brief-COPE scale.

	Active Positive Coping		Behavioral Disengagement		Substance Abuse		Seeking Support	
	β ^+^ (SE ^++^)	*p*	β ^+^ (SE ^++^)	*p*	β ^+^ (SE ^++^)	*p*	β ^+^ (SE ^++^)	*p*
Gender (female vs. male)	0.015 (0.010)	0.124	0.006 (0.016)	0.694	−0.012 (0.009)	0.178	0.018 (0.019)	0.331
Age	−0.001 (0.001)	0.129	0.002 (0.001)	0.021	0.001 (0.001)	0.802	−0.001 (0.001)	0.513
Residence in Greater Attica region (yes vs. no)	0.001 (0.011)	0.932	0.023 (0.017)	0.1720	−0.002 (0.010)	0.835	0.024 (0.021)	0.248
Educational level: middle school–high school vs. primary school	0.011 (0.013)	0.432	0.012 (0.021)	0.56	−0.018 (0.012)	0.123	−0.049 (0.025)	0.050
Educational level: technological educational institute/university vs. primary school	0.026 (0.015)	0.086	−0.025 (0.023)	0.279	−0.009 (0.013)	0.516	−0.047 (0.028)	0.091
Educational level: master’s degree–PhD vs. primary school	0.049 (0.021)	0.020	0.012 (0.033)	0.706	0.006 (0.018)	0.748	−0.078 (0.039)	0.046
Married/living together (yes vs. no)	−0.002 (0.013)	0.890	−0.012 (0.02)	0.540	0.021 (0.011)	0.070	0.002 (0.024)	0.946
Children (yes vs. no)	−0.010 (0.014)	0.461	−0.020 (0.021)	0.360	−0.022 (0.012)	0.071	0.011 (0.026)	0.665
Living alone (yes vs. no)	0.004 (0.015)	0.775	0.006 (0.023)	0.790	0.034 (0.023)	0.069	−0.026 (0.027)	0.344
Employed (yes vs. no)	0.003 (0.014)	0.838	0.038 (0.022)	0.083	0.001 (0.012)	0.974	0.003 (0.026)	0.911
Financial status	0.009 (0.006)	0.161	0.008 (0.010)	0.386	0.016 (0.005)	0.003	0.008 (0.011)	0.493
Months of hemodialysis	0.007 (0.012)	0.57	0.012 (0.019)	0.518	0.003 (0.011)	0.768	0.011 (0.023)	0.621
Vascular access: fistula vs. catheter	−0.006 (0.012)	0.609	−0.013 (0.018)	0.468	0.002 (0.010)	0.872	−0.022 (0.022)	0.298
Vascular access: graft vs. catheter	−0.003 (0.018)	0.876	0.008 (0.028)	0.780	0.010 (0.016)	0.543	−0.01 (0.034)	0.761
Comorbidities (yes vs. no)	0.013 (0.011)	0.232	0.017 (0.018)	0.321	−0.001 (0.010)	0.902	0.025 (0.021)	0.225
Are you a candidate for a kidney transplant? (yes vs. no)	0.005 (0.011)	0.644	0.001 (0.018)	0.980	0.012 (0.010)	0.210	0.01 (0.021)	0.639
Have you had a kidney transplant in the past? (yes vs. no)	0.011 (0.018)	0.558	−0.011 (0.028)	0.700	−0.019 (0.016)	0.229	−0.01 (0.033)	0.758
Emotionality	0.014 (0.006)	0.018	-	-	-	-		
Self-control	-	-	−0.030 (0.009)	0.001	-	-	−0.037 (0.009)	<0.001
Well-being	0.02 (0.005)	<0.001	−0.044 (0.007)	<0.001	−0.012 (0.004)	0.002		
Sociability	-	-	-	-	-	-		
Adjusted R^2^	0.13		0.18		0.15		0.15	
Emotion self-appraisal	-	-	-	-	−0.018 (0.004)	<0.001		
Emotion appraisal of others	-	-	-	-	-	-		
Use of emotion	0.022 (0.004)	<0.001	-	-	-	-		
Regulation of emotion	-	-	−0.037 (0.006)	<0.001	-	-	−0.018 (0.004)	<0.001
Adjusted R^2^	0.13		0.14		0.18		0.13	
	**Religion**		**Humor**		**Avoidance**		**Express negative feelings**	
	**β** **^+^ (SE** **^++^)**	***p***	**β** **^+^ (SE** **^++^)**	***p***	**β** **^+^ (SE** **^++^)**	***p***	**β** **^+^ (SE** **^++^)**	***p***
Gender (female vs. male)	0.089 (0.022)	<0.001	−0.020 (0.020)	0.318	0.048 (0.016)	0.003	0.025 (0.016)	0.119
Age	0.003 (0.001)	0.017	−0.003 (0.001)	0.004	0.001 (0.001)	0.742	0.001 (0.001)	0.404
Residence in Greater Attica region (yes vs. no)	−0.009 (0.024)	0.697	−0.016 (0.022)	0.474	−0.018 (0.017)	0.308	0.024 (0.018)	0.180
Educational level: middle school–high school vs. primary school	0.002 (0.029)	0.947	−0.036 (0.027)	0.177	−0.015 (0.021)	0.474	−0.019 (0.022)	0.382
Educational level: technological educational institute/university vs. primary school	−0.013 (0.033)	0.681	0.001 (0.03)	0.993	−0.04 (0.023)	0.088	−0.013 (0.024)	0.59
Educational level: Master’s degree–PhD vs. primary school	−0.023 (0.045)	0.614	0.001 (0.042)	0.974	−0.017 (0.033)	0.595	−0.034 (0.033)	0.312
Married/living together (Yes vs. No)	−0.042 (0.028)	0.131	0.011 (0.026)	0.659	−0.016 (0.02)	0.436	0.007 (0.021)	0.735
Children (Yes vs. No)	−0.036 (0.03)	0.228	0.034 (0.028)	0.214	−0.009 (0.022)	0.691	−0.009 (0.022)	0.676
Living alone (Yes vs. No)	−0.035 (0.032)	0.267	0.051 (0.029)	0.084	−0.004 (0.023)	0.860	−0.008 (0.023)	0.729
Employed (Yes vs. No)	−0.003 (0.03)	0.912	−0.016 (0.028)	0.569	0.036 (0.022)	0.099	0.043 (0.022)	0.056
Financial status	0.013 (0.013)	0.306	−0.009 (0.012)	0.448	−0.005 (0.009)	0.618	−0.002 (0.01)	0.859
Months of hemodialysis	0.043 (0.026)	0.104	0.008 (0.024)	0.744	−0.025 (0.019)	0.195	−0.022 (0.019)	0.248
Vascular access: fistula vs. catheter	−0.029 (0.025)	0.252	−0.026 (0.023)	0.268	−0.013 (0.018)	0.488	−0.018 (0.019)	0.326
Vascular access: graft vs. catheter	−0.063 (0.04)	0.111	−0.047 (0.036)	0.193	−0.017 (0.029)	0.56	0.013 (0.029)	0.651
Comorbidities (yes vs. no)	0.013 (0.024)	0.596	−0.002 (0.022)	0.934	−0.004 (0.018)	0.83	0.047 (0.018)	0.009
Are you a candidate for a kidney transplant? (yes vs. no)	−0.001 (0.024)	0.974	−0.027 (0.022)	0.23	0.002 (0.018)	0.893	−0.001 (0.018)	0.969
Have you had a kidney transplant in the past? (yes vs. no)	0.028 (0.039)	0.470	−0.039 (0.036)	0.27	0.047 (0.028)	0.098	−0.004 (0.029)	0.885
Emotionality	-	-	-	-	-	-	-	-
Self-control	-	-	-	-	-	-	−0.032 (0.009)	<0.001
Well-being	-	-	0.023 (0.008)	0.007	-	-	−0.019 (0.008)	0.014
Sociability	-	-	-	-	-	-	-	-
Adjusted R^2^	0.15		0.12		0.13		0.17	
Emotion self-appraisal	-	-	-	-	-	-	−0.019 (0.009)	0.048
Emotion appraisal of others	-	-	0.017 (0.008)	0.042	-	-	-	-
Use of emotion	-	-	-	-	-	-	−0.039 (0.008)	<0.001
Regulation of emotion	-	-	-	-	-	-	-	-
Adjusted R^2^	0.15		0.12		0.13		0.16	

^+^ Regression coefficient. ^++^ Standard error. Note 1: Logarithmic transformation of the dependent variable was used for this analysis. Note 2: TEIQue-SF and WEILS values were entered in the analysis alternately due to being highly correlated with each other.

## Data Availability

Data from the present study are not publicly available due to privacy and ethical restrictions but may be shared in anonymized form by the corresponding author upon reasonable request and subject to institutional approval, in accordance with FAIR data principles.
